# Editorial: Meeting of the Portuguese Society for Neurosciences SPN2019

**DOI:** 10.3389/fnmol.2021.753500

**Published:** 2021-10-07

**Authors:** Maria José Diógenes, Cláudia Guimas Almeida, Sara Xapelli

**Affiliations:** ^1^Faculdade de Medicina, Instituto de Medicina Molecular João Lobo Antunes, Universidade de Lisboa, Lisbon, Portugal; ^2^Faculdade de Medicina, Instituto de Farmacologia e Neurociências, Universidade de Lisboa, Lisbon, Portugal; ^3^iNOVA4Health, Chronic Diseases Research Center (CEDOC), NOVA Medical School (NMS), Universidade Nova de Lisboa, Lisbon, Portugal

**Keywords:** neurodevelopment, neurodegeneration, glia and neuroinflammation, drug and addiction, sensory processing, rare disorders, cellular and molecular neurosciences

The Portuguese Society for Neuroscience meeting (SPN2019) was held in Lisbon from 30th May to 1st June, 2019. The main purpose of this meeting was to promote and develop research in Neurosciences. This interaction took place in a privileged way at this meeting and brought together the vibrant Portuguese Neuroscience community that significantly advances this defying area.

This Research Topic comprises nine manuscripts covering some of the work presented at SPN2019 and hot topics in neurosciences: neurodevelopment; cellular and molecular neurosciences; neurodegeneration; glia and neuroinflammation; drug and addiction; sensory processing; and rare disorders.

In a brief Research Report, Miranda et al. show that cannabidiol, Δ9-tetrahydrocannabinol, and two synthetic cannabinoids (THJ-018 and EG-018) profoundly impact, from toxicity to precocity, developing neurons, highlighting the negative effect of prenatal exposure to natural and synthetic cannabinoids.

A brief Research Report by Neiva et al. shows that methylprednisolone differentially affects GABA and glutamate release from rat hippocampal nerve terminals *via* fast non-genomic mechanisms putatively involving the activation of membrane-bound corticosteroid receptors.

Original Research by Sa de Almeida et al. reveals that microglial Sirtuin 2 (Sirt2) prevents NMDA-mediated excitotoxicity in hippocampal slices in response to an inflammatory signal. Overall, the data suggest a key-protective role for microglial Sirt2 in amnesic deficits associated with neuroinflammation.

Original Research by Fonteles et al. supports that antagonists of ATP receptors P2X7 are protective of dyskinesia induced by dopamine replacement therapy in a Parkinson's disease rat model. These findings suggest P2X7 antagonists as novel candidate anti-dyskinesia drugs.

Original Research by Costa-Pereira et al. describes an enhancement of the descending noradrenergic pain control system due to neuropathy induced by cancer chemotherapy. The authors suggest that potentiation of the antinociception mediated by alpha2-adrenoreceptors may be a therapeutic opportunity.

Original Research by Fabricio de Sousa et al. shows that the cerebellum is vulnerable to metabolic changes due to ultra-endurance racer. High-volume training in rodents induced cerebellar oxidative and inflammatory status and impaired astrocyte reactivity.

Mouro et al. reviewed the recent evidence concerning metabolic, synaptic, functional, and molecular dysfunctions occurring in Rett syndrome. From modulators of GABAergic signaling to cannabinoids and the ketogenic diet, cleverly exploiting the metabolic features of this disease, an ample bulk of evidence has been gathered, creating a plethora of research lines to be followed in the future.

Perdigão et al. reviewed the intracellular trafficking mechanisms of synaptic dysfunction in Alzheimer's disease. They described that in early and late-onset familial Alzheimer's disease the earliest synaptic dysfunctions are characterized by disruptions of the presynaptic vesicle exo- and endocytosis and postsynaptic glutamate receptor endocytosis. While in early-onset familial Alzheimer's disease, synapse dysfunction seems to be triggered by Aβ, in late-onset Alzheimer's disease, there might be a direct synaptic disruption by late-onset Alzheimer's disease trafficking genes, requiring further research.

The mini-review by Gonçalves-Ribeiro et al. discussed the role of the increase in glutamate uptake on hippocampal long-term potentiation and depression beyond excitotoxicity.

Overall, this Research Topic compiling both original and review papers shows the diversity in the neuroscience field ranging from *in vitro* to *in vivo* approaches, including rare to common disorders, neurodevelopment to aging, and molecular to behavioral tests. We hope this collection of articles will be highly attractive to the international neuroscience community.

## Author Contributions

All authors listed have made a substantial, direct and intellectual contribution to the work, and approved it for publication.

## Conflict of Interest

The authors declare that the research was conducted in the absence of any commercial or financial relationships that could be construed as a potential conflict of interest.

## Publisher's Note

All claims expressed in this article are solely those of the authors and do not necessarily represent those of their affiliated organizations, or those of the publisher, the editors and the reviewers. Any product that may be evaluated in this article, or claim that may be made by its manufacturer, is not guaranteed or endorsed by the publisher.

